# Outdoor Scene Understanding Based on Multi-Scale PBA Image Features and Point Cloud Features

**DOI:** 10.3390/s19204546

**Published:** 2019-10-19

**Authors:** Yisha Liu, Yufeng Gu, Fei Yan, Yan Zhuang

**Affiliations:** 1 Information Science and Technology College, Dalian Maritime University, Dalian 116026, China; liuyisha@dlmu.edu.cn; 2 School of Control Science and Engineering, Dalian University of Technology, Dalian 116024, China; guyufeng@mail.dlut.edu.cn (Y.G.); zhuang@dlut.edu.cn (Y.Z.)

**Keywords:** 3D point cloud, outdoor scene understanding, mobile laser scanning

## Abstract

Outdoor scene understanding based on the results of point cloud classification plays an important role in mobile robots and autonomous vehicles equipped with a light detection and ranging (LiDAR) system. In this paper, a novel model named Panoramic Bearing Angle (PBA) images is proposed which is generated from 3D point clouds. In a PBA model, laser point clouds are projected onto the spherical surface to establish the correspondence relationship between the laser ranging point and the image pixels, and then we use the relative location relationship of the laser point in the 3D space to calculate the gray value of the corresponding pixel. To extract robust features from 3D laser point clouds, both image pyramid model and point cloud pyramid model are utilized to extract multiple-scale features from PBA images and original point clouds, respectively. A Random Forest classifier is used to accomplish feature screening on extracted high-dimensional features to obtain the initial classification results. Moreover, reclassification is carried out to correct the misclassification points by remapping the classification results into the PBA images and using superpixel segmentation, which makes full use of the contextual information between laser points. Within each superpixel block, the reclassification is carried out again based on the results of the initial classification results, so as to correct some misclassification points and improve the classification accuracy. Two datasets published by ETH Zurich and MINES ParisTech are used to test the classification performance, and the results show the precision and recall rate of the proposed algorithms.

## 1. Introduction

Outdoor scene understanding based on mobile laser scanning (MLS) point cloud data are a fundamental ability for unmanned vehicles and autonomous mobile robots navigating in urban environments. Recently, a variety of laser point cloud processing methods have been presented to recognize the main elements of road environment [1], to accomplish robust place recognition [2], to extract parameters of trees [3], and so on. Moreover, the point clouds obtained from a laser scanner can also be utilized to accomplish real-time shape acquisition [4], outdoor 3D laser data classification [5], and outdoor scene understanding [6]. A state-of-the-art review for object recognition, segmentation, and classification of MLS Point Clouds was also given in [7].

In order to reduce the computational complexity of feature extraction and classification, some scholars have converted 3D laser point clouds into 2D images and used image processing methods to process 3D point clouds, such as range image [8], reflectance image [9], and bearing angle image (BA image) [10]. The BA image was originally used to solve the calibration problem between camera and laser scanner [10]. Since the BA image has clearer texture details than range image and reflectance image, it is also used to solve the laser point cloud classification problem in outdoor or indoor scenes. Zhuang et al. used a 2D BA image to represent the outdoor 3D point cloud [11]. By extracting texture features from the BA image, the scene understanding of the 3D point cloud was realized. Zhang et al. studied the problem of 3D object detection in a cluttered indoor environment and transformed the 3D laser point cloud into a 2D BA image, which enabled the robot to complete the task of scene understanding at a lower computational cost [12]. However, the quality of the BA image depends on the selection of the viewpoint position. If the viewpoint is not selected properly, the image will be indistinct. In addition, the BA image also has the problem of grayscale change for the same object.

It is very important to determine the neighborhood range of laser points for feature extraction [13]. There are two kinds of neighborhood selection methods: fixed-scale neighborhood selection and multi-scale neighborhood selection. Fixed-scale neighborhood selection depends heavily on experience. If the scale is too small, the whole view cannot be seen clearly. If the scale is too large, the details can easily be ignored [14]. Therefore, the multi-scale feature extraction of 3D point clouds is a good choice, which is very helpful for improving classification performance.

Many classifiers can be used for point cloud classification, such as Support Vector Machine classifier, Nearest Neighbor classifier, K Nearest Neighbor classifier, and Naive Bayesian classifier [15]. When the selected feature dimension is very high (over 100 dimensions) and there are a lot of redundant features, the Random Forest classifier can process the high-dimensional data and complete the feature screening [16]. In addition, some classification methods based on contextual information of the point cloud are also widely used. Munoz et al. proposed a point cloud classification framework based on the Markov Random Field. Based on the contextual information, the classification of vehicular laser point cloud was realized [17]. Najafi et al. introduced a non-associative higher-order Markov Random Field to address the problem of semantic 3D point classification, which took into account the non-associative geometric context between different classes [18].

In this paper, a novel image model named PBA image is firstly proposed to represent the MLS point cloud data, which shows superior performance to display a large-scale scene with a panoramic view. Compared with the traditional BA model, the PBA model can still transform the unordered laser scanning data to a 2D image without fixed scan sequence relationships. To improve the accuracy and robustness of scene understanding results, multiple-scale features are extracted not only from the PBA images but also from the corresponding original LiDAR point clouds. In our work, the Random Forest algorithm is adopted to build the classifier which can complete feature screening and improve the generalization ability of classification. After the initial classification, superpixel segmentation is performed on the PBA images, which considers the contextual information between laser points in 2D images. Within each superpixel block, reclassification algorithm is performed based on the results of the initial classification, so as to correct partial misclassification points. A series of experimental results from both ETH Zurich and MINES ParisTech datasets are given to test the validity and robustness of the proposed approach.

## 2. Panoramic Bearing Angle Images Generating from 3D Laser Point Clouds

**Figure 1:**
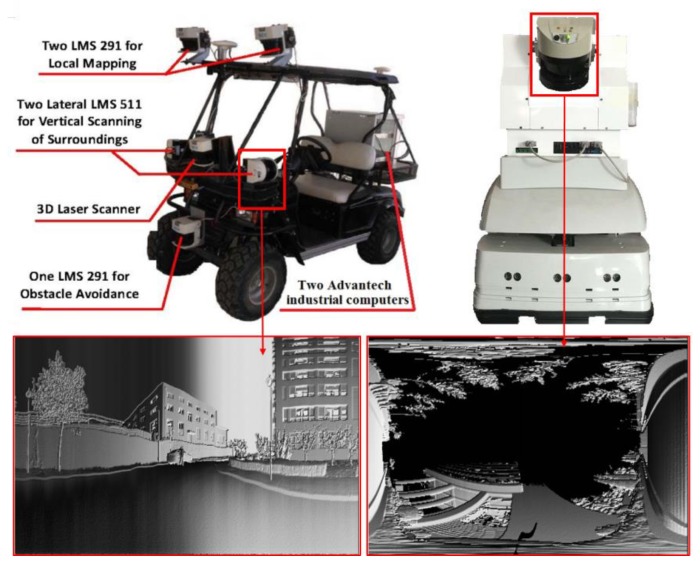
Grayscale images generated from laser scanning points obtained by the 2D laser scanners equipped on mobile robots.

Generally speaking, there are two ways to obtain 3D laser point clouds to represent large-scale outdoor scenes in the field of mobile robotics. The first one is to install a 2D laser scanner on a mobile robot to perform on-the-fly scanning or fixed-point scanning. As shown in Figure 1, the left one is a driverless car using two lateral SICK LMS 511 laser ranger finders (produced by SICK AG, Waldkirch, Germany) to capture groups of sequenced 2D laser points in the on-the-fly scanning mode, while the right one is a mobile robot using a pitch rotating SICK LMS 511 to obtain 3D point clouds in fixed-point scanning mode. In these cases, the 3D point clouds are composed of sequential 2D laser scanning points and can be represented by a matrix, which can be transformed to grayscale images by using the Bearing Angle (BA) image model.

The other method is to use the 3D laser scanner to obtain the 3D point clouds directly. However, these 3D point clouds are composed of several groups of scanning data and are always unordered when stored, so they cannot be represented by a matrix. In addition, in most public laser scanning datasets, there are no scan sequence relationships stored between different laser scans. To solve this problem, a novel Panoramic Bearing Angle (PBA) image model is proposed in this paper and introduced as follows.

### 2.1. Projection of 3D Laser Point Cloud to Pixel Plane

**Figure 2:**
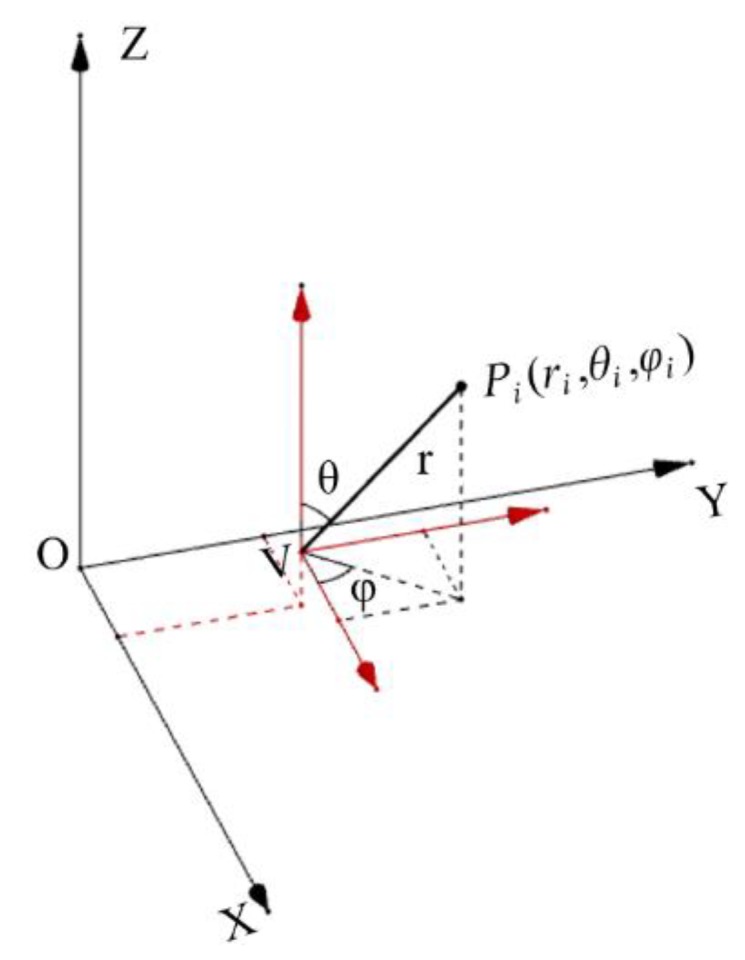
An illustration of coordinate transformation.

Viewpoint selection is a crucial step for 2D images generating from 3D laser point clouds. For fixed-point scanning, the location of the rotating 2D laser range finder is selected as the viewpoint. For on-the-fly scanning, the viewpoint is usually selected on the trajectory of the moving laser range finder. Suppose that a selected viewpoint of a 3D point cloud is *V*(*x*_v_,*y*_v_,*z*_v_), a laser point in the cloud is *P_i_* (*x_i_*,*y_i_*,*z_i_*), and the matrix size for the 2D image to be generated is *M*×*N*. As shown in Figure 2, a spherical coordinate system is established in which the viewpoint *V* is the center of the sphere. It should be noted that the size of the panoramic image is only related to the resolution of the image (the size of the image matrix) regardless of the size of the projection surface. According to (1), the original 3D laser point *P_i_* (*x_i_*,*y_i_*,*z_i_*) is converted from the global coordinate system to the spherical coordinate system with the viewpoint *V* as the center of the sphere. The point in the spherical coordinate system is *P_i_*(*r_i_*,*θ_i_*,*φ_i_*).

(1) $$\{\begin{array}{c} r_{i}=\sqrt{{\left(x_{i}-x_{v}\right)}^{2}+{\left(y_{i}-y_{v}\right)}^{2}+{\left(z_{i}-z_{v}\right)}^{2}}\\ \theta_{i}=\arccos(\frac{z_{i}-z_{v}}{r_{i}})\;\\ \varphi_{i}=\arctan(\frac{y_{i}-y_{v}}{x_{i}-x_{v}})\; \end{array},$$

where $\theta_{i}\in [0,\pi ]\;\mathrm{and}\;\varphi_{i}\in [0,2\pi ]$.

**Figure 3:**
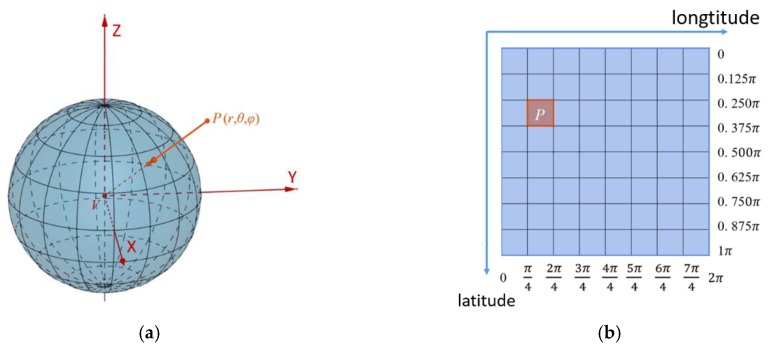
An illustration of laser ranging point projection. (**a**) A spherical coordinate system; (**b**) 2D matrix of the PBA.

According to (2), *M* warps *l_m_* and *N* + 1 wefts *l_p_* are drawn which can divide the sphere into *M × N* independent grids. The left image in Figure 3 is a spherical coordinate system which is divided into 64 grids by eight warps and nine wefts (two poles are included).

(2) $$\{\begin{array}{l} l_{m}=m\times \frac{2\pi }{M},\;m\in [0,M-1]\\ l_{p}=p\times \frac{\pi }{N},\;p\in [0,N]\; \end{array}$$

Take the center of the sphere *V* as the starting point and make a ray through each laser scanning point *Pi(r_i_,θ_i_,φ_i_)*, so that the laser point can be projected to a grid of the sphere. If there are more than one projections of laser points in a grid, the one closest to the center of the sphere is retained. Then cut the spherical surface along the 0-degree warp and spread it to the horizontal plane to obtain the 2D matrix of the PBA (see the right image of Figure 3).

**Figure 4:**
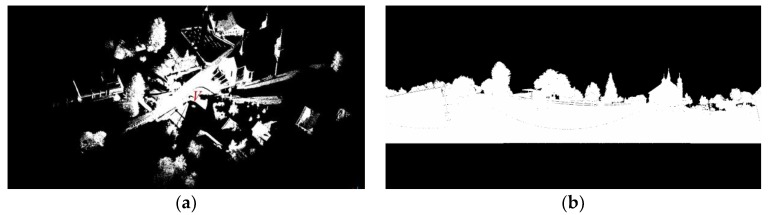
(**a**) A whole view of a large-scale outdoor scene represented by the original 3D point cloud; (**b**) The panoramic binarized image of the same scene’s point cloud projected to the spherical coordinate system.

As shown in Figure 4a, a 3D laser point cloud is obtained in the fixed scanning point *V*, and Figure 4b is the corresponding panoramic image, which is displayed in the binary value. The white pixel indicates that there is a laser scanning point corresponding to it, while the black pixel indicates that no laser point corresponds to it.

### 2.2. Calculating of Image Gray Value

**Figure 5:**
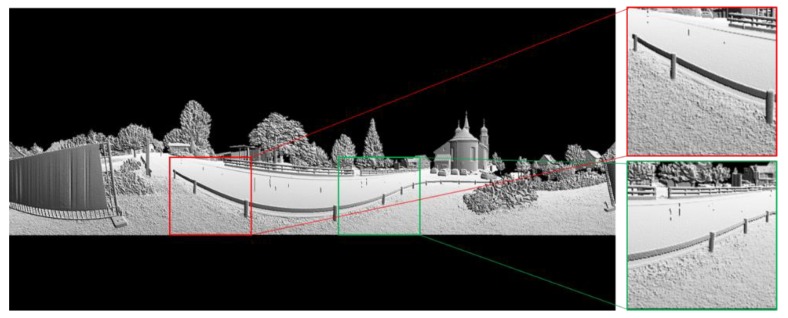
An example of a Bearing Angle (BA) image for an outdoor scene.

There are many classical image models to represent laser points stored in the 2D matrix, such as reflectance image, range image and bearing angle (BA) image. However, the reflectance image is less robust and the edge description in range image is not clear enough, especially in large-scale scenes. The quality of the BA image depends on the selection of the viewpoint position. In addition, grayscale change may appear in the BA image. As shown in Figure 5, the gray values for the same railing are inconsistent, which is not beneficial to feature extraction and classification.

In order to overcome the above limitations, a novel PBA image model is proposed in this paper inspired by the BA model, which is not related to the selection of viewpoints. Moreover, the PBA image model can provide stable gray values for the same object and also ensure clear texture and high image contrast with high computational efficiency.

**Figure 6:**
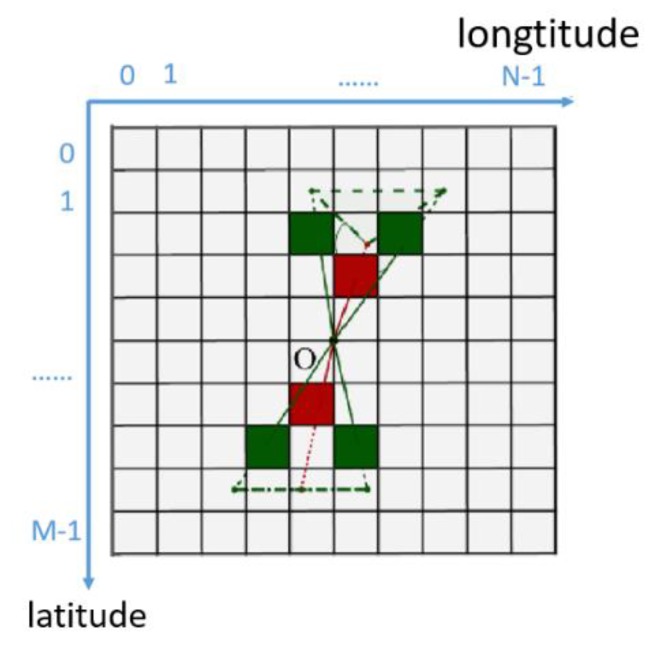
An illustration of the grayscale calculation.

Here we will explain how to calculate the gray value of each pixel in the PBA image. As shown in Figure 6, there are *M* rows in the image matrix, and the image pixel corresponding to the laser scanning point *P* is defined as *P_x,y_*, which is located in row x and column *y*. Two neighboring laser points *P_l_* and *P_r_* for point *P* are chosen as:

(3) $$\{\begin{array}{l} \begin{array}{l} \Psi (P_{l})=P_{x-1,y-1},\;\Psi (P_{r})=P_{x-1,y+1},\;X<M/2\\ \Psi (P_{l})=P_{x+1,y-1},\;\Psi (P_{r})=P_{x+1,y+1},\;X\geq M/2 \end{array} \end{array}$$

where Ψ(**∙**) represents the image pixel of a laser point. If the pixel is in the upper part of the image, its upper left and upper right pixels are selected as neighboring pixels; otherwise, the lower left and right lower pixels are selected as neighboring pixels.

The pixel gray value of *P_x,y_* is defined as:

(4) $$G_{X,Y}=255(1-\alpha /2\pi )$$

where *α* is the angle between *P* and its neighboring laser scanning points *P_l_* and *P_r_*, which can be obtained as follows:

(5) $$\{\begin{array}{ll} \alpha =∠P_{l}PP_{r}, & \left|VP\right|\geq (\left|VP_{l}\right|+\left|VP_{r}\right|)/2\\ \alpha =2\pi -∠P_{l}PP_{r}, & \left|VP\right|<(\left|VP_{l}\right|+\left|VP_{r}\right|)/2 \end{array}$$

where *VP, VP_l_*, *VP_r_* represent the distances between the center of the sphere *V* and laser points *P, P_l_*, *P_r_*, respectively.

**Figure 7:**
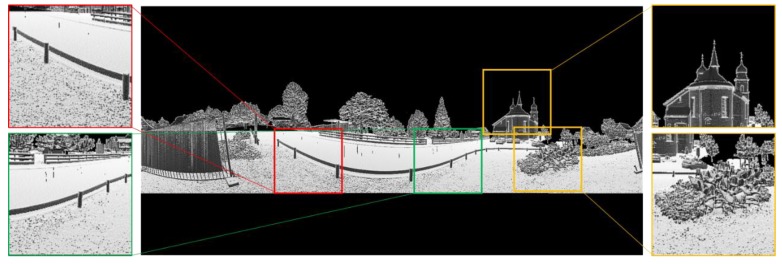
An example of a Panoramic Bearing Angle (PBA) image for the same outdoor scene.

An example of a PBA gray image is given in Figure 7. Compared with the BA image in Figure 5, the gray values for the same railing are consistent, and the boundaries of the objects in the scene are clearer.

## 3. Laser Point Clouds Classification Using Multi-Scale PBA Image Features and Point Cloud Features

It is important to select the neighborhood range of the laser points in the feature extraction step. In our work, the image pyramid model is adopted to extract the texture features of PBA images on multiple scales. The point cloud pyramid model is then used to extract the local features of the 3D point cloud on multiple scales.

### 3.1. Multi-Scale PBA Image Feature Extraction

**Figure 8:**
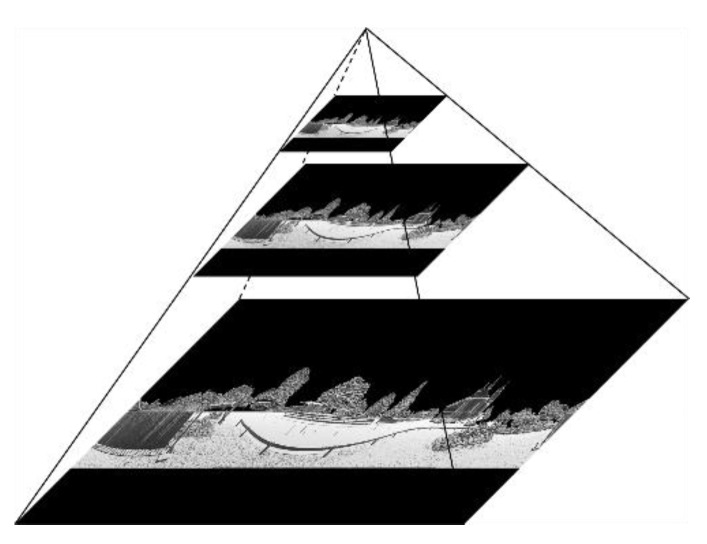
An example of image pyramid model for PBA image.

In our work, feature extraction is accomplished in 2D gray images on multiple scales. When the scale is large, the computational cost is very high. Therefore, the PBA image is downsampled by using the image pyramid model [19]. The image pyramid model for PBA images is given in Figure 8. It should be noted that the image in each layer of the pyramid model is generated directly from the 3D laser point cloud, rather than from the downsampling of the original image.

**Figure 9:**
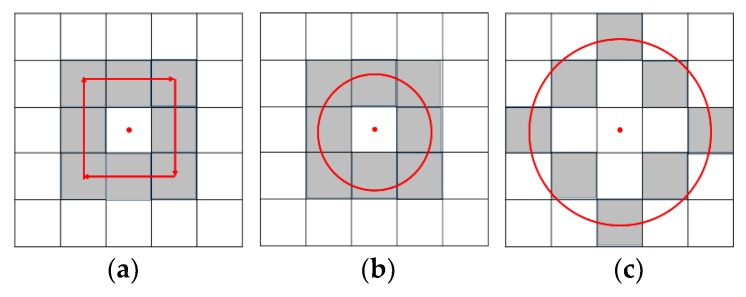
(**a**) Classic neighborhood selection method for classic LBP features; (**b**) Improved neighborhood selection method for LBP features (r = 1); (**c**) Improved neighborhood selection method for LBP features (r = 2).

Local Binary Pattern (LBP) is a kind of image texture feature, which is extracted from multi-resolution PBA images. For the classic LBP feature, eight fixed neighborhood pixels are selected (see Figure 9a). In order to extract multi-scale texture features an improved neighborhood selection method [20] is adopted for LBP feature extraction in our work, in which a circular neighborhood is selected with variable radius *r*. The pixel coordinate of the neighborhood points (*x_p_*, *y_p_*) can be obtained as follows:

(6) $$\{\begin{array}{c} x_{P}=x_{c}+r\times \cos(\frac{2\pi P}{8})\\ y_{P}=y_{c}-r\times \sin(\frac{2\pi P}{8}) \end{array},\;P=0,1,\cdots 7$$

where (*x_c_*, *y_c_*) is the pixel coordinate of the center pixel. As shown in Figure 9a,b, *r* is selected as 1 and 2, respectively.

Reduce the original 256-level gray PBA image to 3-level and the pixel gray value *G_new_* of the simplified 3-level image can be obtained as follows:

(7) $$G_{new}=\{\begin{array}{cc} 0\;, & G_{old}=0\\ 127, & 1\leq G_{old}\leq 127\\ 255, & 128\leq G_{old}\leq 255 \end{array}$$

where *G_old_* is the pixel gray value of the original PBA image. 

**Figure 10:**
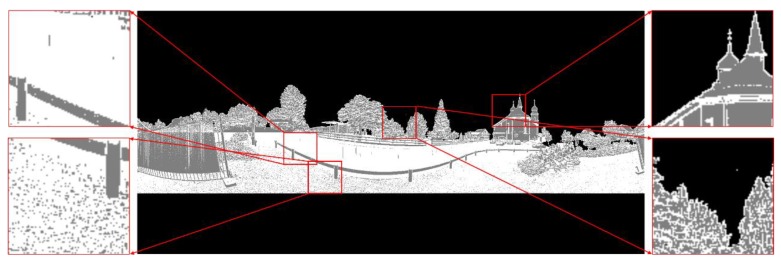
An example of a 3-level gray PBA image.

Figure 10 shows an example of the simplified 3-level PBA image (black-0; gray-127; white-255), and four categories of typical local scenes also show distinct texture features, which are artificial ground (top left), natural ground (bottom left), buildings (top right), and vegetation (bottom right). 

When feature extraction in different layers of the image pyramid model for the PBA images is completed, these features in different layers need to be fused. Starting from the top layer image of the pyramid, the image features are upsampled, and then superimposed with the image features of the next layer. These two steps are repeated until the features in all layers are superimposed on the image at the bottom layer of the image pyramid model.

In summary, the (*P* + 1) layer image pyramid model of PBA images is built from the original laser point cloud, and each layer of PBA images is converted to a 3-level gray image. LBP features are then extracted in each image pixel on *m* scales. Finally, the features in different layers are superimposed together from the top layer to the bottom layer. Therefore, there are *m* × (*P* + 1) image features for every pixel in the original PBA image.

### 3.2. Multi-Scale Point Cloud Feature Extraction

In our work, features are extracted from 3D laser point clouds on multiple scales. However, when the neighborhood radius is expanded at a linear rate, the number of neighborhood points of a laser point is approximately increased at a cubic speed, which greatly increases the computational burden. In order to solve this problem, the point cloud pyramid model is derived which is inspired by the image pyramid model in image processing.

**Figure 11:**
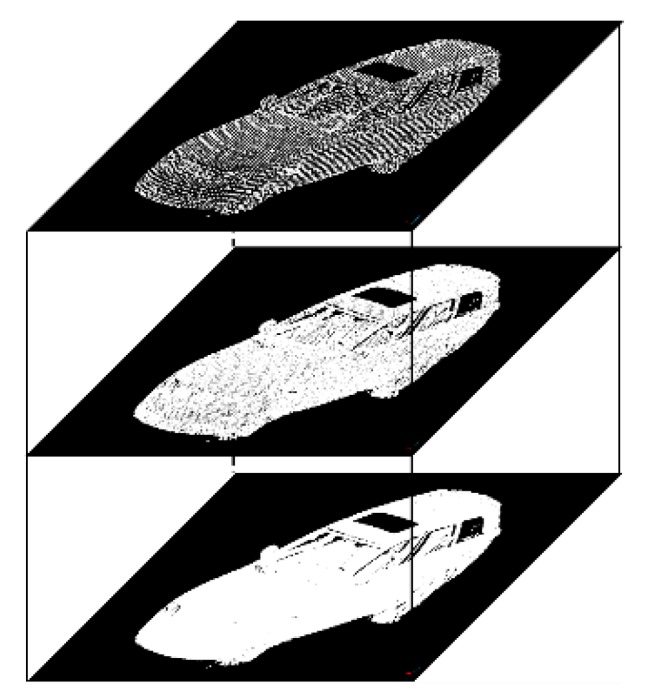
An illustration of point cloud pyramid model.

Similar to the image pyramid model, the downsampling algorithm is utilized for the original point clouds to build the point cloud pyramid model. The voxel model is used to divide the laser point cloud to be downsampled into different 3D grids. Then the center of gravity of the laser points in each voxel (3D grid) is calculated to represent all the points in the voxel. An illustration of the point cloud pyramid model is shown in Figure 11, in which the bottom layer is the original laser point cloud. Then a fixed number of laser points are selected as neighborhood points in different layers of the point cloud pyramid model.

After determining the neighborhood range of each laser point, feature extraction will be performed which includes statistical features, geometric morphological features, and histogram features.

#### 3.2.1. Statistical Features

Let the total number of laser points in the current neighborhood be (*k* + 1), and the coordinate of the lowest point in the neighborhood be *h*_min_. In our work, five statistical features are extracted, which are:

- *h*, the absolute height of the laser point;
- $\Delta h=h-h_{\min}$, the relative height between the laser point and the lowest laser point in the neighborhood;
- $\sigma =\sqrt{\frac{1}{k}\sum_{i=1}^{k}\left(h_{i}-\bar{h}\right)}$, the standard deviation of the laser point’s height in the neighborhood;
- *r*, the radius of the maximum bounding sphere of the neighborhood;
- $d=\frac{k+1}{\frac{4}{3}\pi r^{3}}$, the density of the laser points in the neighborhood.

#### 3.2.2. Morphological Features

According to the summary in [15], a covariance matrix is adopted to describe the 3D laser point cloud in the neighborhood, where *p_c_* is the current query point and *p_i_* is the neighborhood point around the query point. The covariance matrix can be expressed as:

(8) $$\mathrm{COV}=\frac{1}{k}\sum_{i=1}^{k}\left(p_{i}-p_{c}\right){\left(p_{i}-p_{c}\right)}^{T}$$

which is a three-dimensional positive definite matrix. By eigen decomposition, three eigenvalues *λ*_1_, *λ*_2_, *λ*_3_ (let *λ*_1_ ≥ *λ*_2_ ≥ *λ*_3_ ≥ 0) and three eigenvectors *e*_1_, *e*_2_, *e*_3_ corresponding to *λ*_1_, *λ*_2_, *λ*_3_ are obtained, respectively. In our work, nine morphological features are extracted, which are Linearity *L_λ_*, Planarity *P_λ_*, Sphericity *S_λ_*, Omnivariance *O_λ_*, Anisotropy *A_λ_*, Eigenentropy *E_λ_*, Sum Σ*_λ_*, Change of Curvature *C_λ_* and Verticality *V_λ_*. These features can be calculated as follows:

(9) $$L_{\lambda }=\frac{\left(\lambda_{1}-\lambda_{2}\right)}{\lambda_{1}},\;P_{\lambda }=\frac{\left(\lambda_{2}-\lambda_{3}\right)}{\lambda_{1}},\;S_{\lambda }=\frac{\lambda_{3}}{\lambda_{1}},\;O_{\lambda }={\left(\lambda_{1}\cdot \lambda_{2}\cdot \lambda_{3}\right)}^{\frac{1}{3}},\;A_{\lambda }=\frac{\left(\lambda_{1}-\lambda_{3}\right)}{\lambda_{1}},\;\;E_{\lambda }=-\sum_{i=1}^{3}\lambda_{i}\ln(\lambda_{i}),\;\Sigma_{\lambda }=\lambda_{1}+\lambda_{2}+\lambda_{3},\;C_{\lambda }=\frac{\lambda_{3}}{\lambda_{1}+\lambda_{2}+\lambda_{3}},\;V_{\lambda }=1-e_{z}$$

#### 3.2.3. Histogram Features

**Figure 12:**
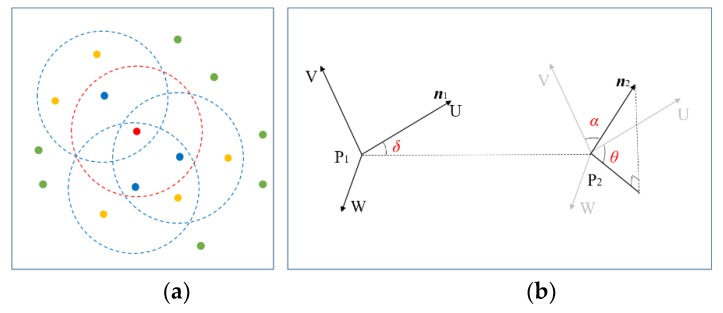
An illustration of FPFH feature extraction. (**a**) FPFH consists of two Simplified Point Feature Histograms (SPFH); (**b**) The position relationship between two laser points.

Fast point feature histograms (FPFH) is a set of 33-dimensional histogram features [21]. Compared to morphological features, FPFH can describe the geometric features in the query point’s neighborhood in more detail and represent the roughness of the plane effectively, which can be used to distinguish two typical road surfaces (artificial ground and natural ground). As shown in Figure 12a, FPFH consists of two Simplified Point Feature Histograms (SPFH). One is composed of the query point *p* and its neighborhood point *p_k_* (the points in the red circle) and the other one is composed of each neighborhood point *p_k_* and its neighborhood points (the points in the blue circle). FPFH can be defined as follows:

(10) $$\mathrm{FPFH}(p)=\mathrm{SPFH}(p)+\frac{1}{k}\sum_{i=1}^{k}\frac{1}{w_{k}}\cdot \mathrm{SPFH}(p_{k})$$

where *k* stands for the number of neighborhood points around the query point *p*, *w_k_* stands for distance weight which is used to measure the density between neighborhood points and query points.

SPFH is composed of Simplified Point Features (SPF). SPF is a three-dimensional angular feature descriptor that represents the position relationship between two laser points. As shown in Figure 12b, P_2_ is a laser point in the neighborhood of P_1_, and ***n***_1_ and ***n***_2_ are the normal vectors of P_1_ and P_2_. According to (12), the UVW coordinate system is established with P1 as the coordinate origin:

(11) $$\{\begin{array}{l} u={\vec{n}}_{1}\\ v=u\times \frac{\left(p_{2}-p_{1}\right)}{{\left\|p_{2}-p_{1}\right\|}_{2}}\\ w=u\times v \end{array}$$

The angular parameter *δ*, *α*, *θ* are used to describe the position relationship between two laser points, which can be defined as follows:

(12) $$\{\begin{array}{l} \alpha =v\cdot {\vec{n}}_{2}\\ \delta =u\cdot \frac{\left(p_{2}-p_{1}\right)}{{\left\|p_{2}-p_{1}\right\|}_{2}}\\ \theta =\arctan(w\cdot {\vec{n}}_{2},\;u\cdot {\vec{n}}_{2}) \end{array}$$

Although FPFH can describe the geometric characteristics of the laser point cloud in more detail, it increases the computational burden significantly. Therefore, we only extract FPFH features for the laser point at the bottom of the point cloud pyramid, while the other 14-point cloud features (five statistical features and nine morphological features) are extracted for each laser point of the point cloud pyramid.

### 3.3. Classification with Random Forest and Reclassification Based on the Contextual Information

In this paper, the Random Forest classifier is used to perform feature screening on the extracted high-dimensional features, and the initial classification of the 3D laser point clouds is implemented. Since this method does not consider the contextual information between laser points, the credibility of classification results is low for the objects with similar local features (such as eaves and vegetation). In order to make full use of the contextual information between laser points, the classification results are remapped into the PBA images, and superpixel segmentation is performed on the PBA images. Within each superpixel block, the classification is performed again based on the results of the initial classification, so as to correct partial misclassification points and further improve the classification accuracy.

The Random Forest classifier is composed of multiple decision tree classifiers. In the training stage, some training samples are randomly selected to complete the training for each decision tree. In the classification stage, some decision trees are randomly selected and the mode of their output categories is taken as the final classification result. 

**Figure 13:**
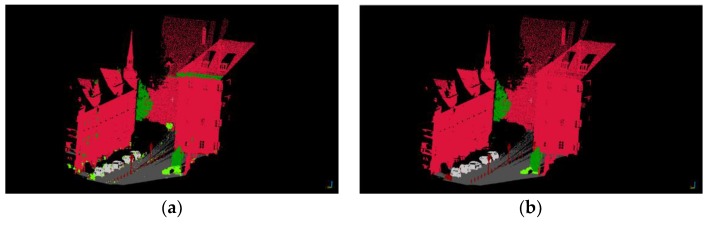
Classification result (**a**) and the ground truth (**b**).

Figure 13 shows the classification results by using the Random Forest classifier and the ground truth. Seven different colors are used to represent seven different categories: dark gray for artificial ground, yellow for natural ground, dark green for high vegetation, light green for low vegetation, red for buildings, dark brown for railings, and silver for cars. From the classification results, we can see that the main objects, such as buildings, ground, cars, and vegetation, can be effectively classified. 

By comparing the classification results with the ground truth, we can find that a large number of laser points that do not belong to vegetation are classified into vegetation. This is due to the cluttered distribution of these laser points, and the local features of these laser points are very close to those of the vegetation. Therefore, the laser point clouds will be reclassified by considering the contextual information of the 3D laser point clouds based on the PBA images.

In this paper, the SEEDS-based superpixel segmentation is performed on the PBA images [22]. For each superpixel block, if the pixel proportion of vegetation is less than a threshold, the laser points corresponding to vegetation will be reclassified into the category with the highest pixel proportion in the block. This strategy makes full use of the contextual information of the 3D laser point cloud in 2D images, which can reduce the error rate of the point cloud classification

**Figure 14:**
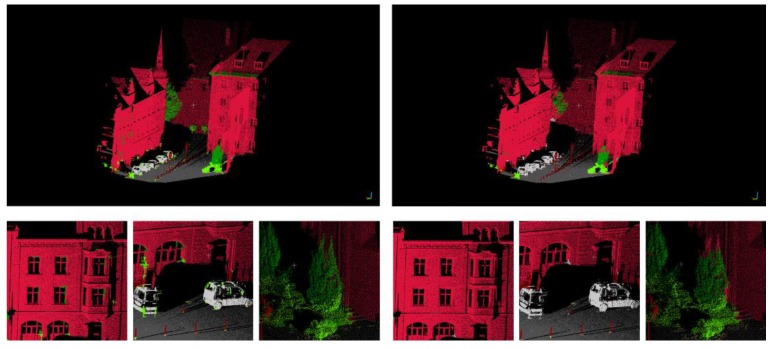
Initial classification result (**top left**) and reclassification result (**top right**); local details of the initial classification result (**bottom left**) and local details of reclassification result (**bottom right**).

As shown in Figure 14, the initial classification result based on the Random Forest classifier is at the top left and the reclassification result is at the top right. The bottom left images and the bottom right images are local details in enlarged images of the initial classification result and reclassification result, respectively. After reclassification, most of the point clouds that were previously misclassified are corrected. 

## 4. Experimental Results

### 4.1. Classification Results of 3D Point Clouds Obtained in Fixed-Point Scanning Mode

In this subsection, a 3D laser point cloud dataset published by ETH Zurich is selected to verify the algorithm. This dataset includes 15 typical scenes. Two typical scenes are selected for testing, and the remaining scenes are used for training. The two testing sets contain seven categories which are represented by seven different colors: dark gray for artificial ground, yellow for natural ground, dark green for high vegetation, light green for low vegetation, red for buildings, dark brown for railings, and silver for cars. The category distribution of the two testing sets is shown in Table 1.

**Figure 15:**
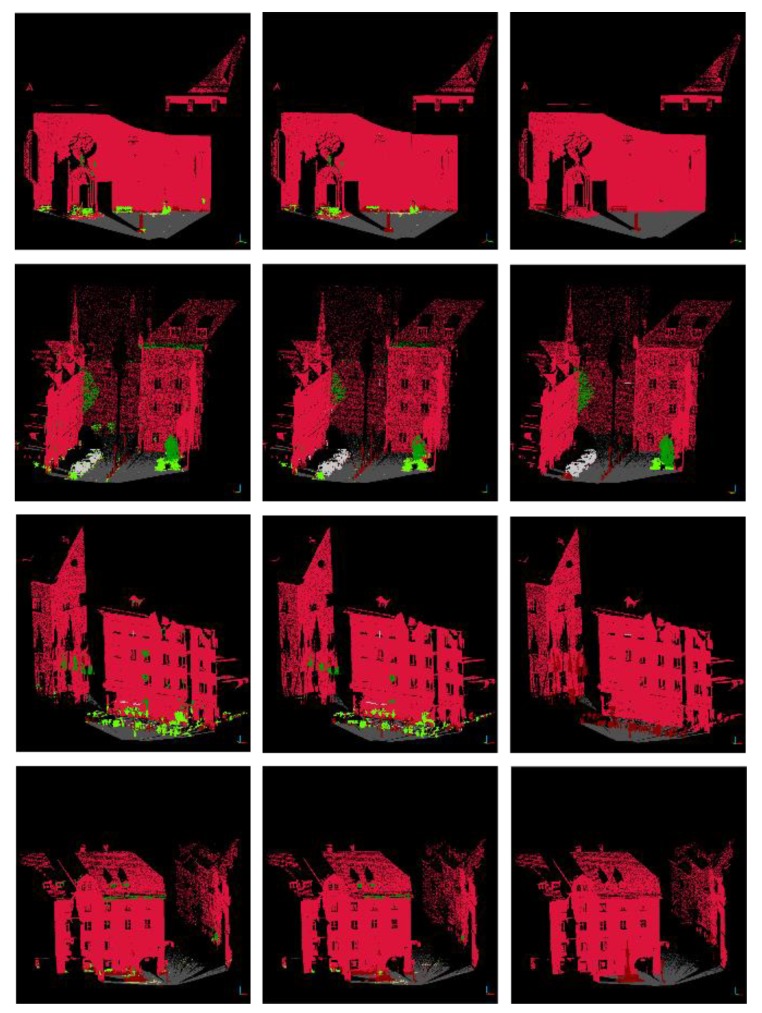
The classification results for Testing Set A (initial classification results (**left**); reclassification results (**middle**); ground truth (**right**)).

**Figure 16:**
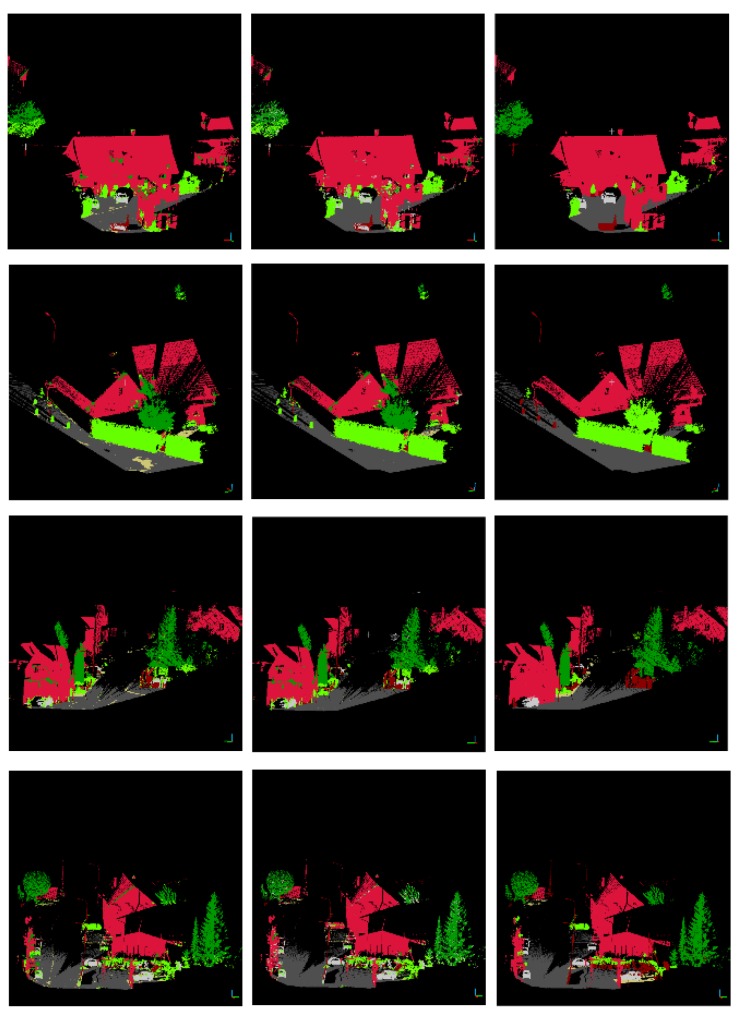
The classification results for Testing Set B (initial classification results (**left**); reclassification results (**middle**); ground truth (**right**)).

A four-layer image pyramid model and a six-layer point cloud pyramid model are established. The resolution of the bottom image is 1440 × 720 and the density of the bottom point cloud is 25^3^ points/m^3^. For the image pyramid model, the texture features are extracted at six scales. For the point cloud pyramid model, the 10 and 20 nearest laser points are selected respectively as neighborhood points for each laser query point. The 14-point cloud features (five statistical features and nine morphological features) are extracted for each laser query point. Thirty-three FPFH features are only extracted for the laser point at the bottom of the point cloud pyramid. Therefore, for each laser point, 24 (4 × 6) image texture features and 201 (6 × 2 × 14 + 33) point cloud features are extracted. The Random Forest classifier consists of 200 decision trees with a depth of 15. The images on the left of Figure 15 and Figure 16 show the initial classification results. The recall rate and precision rate are given in Table 2 and Table 3, respectively.

According to the initial classification results, it can be seen that the recall rates of vegetation and natural ground are very low. A large number of laser points that belong to cars and buildings are misclassified into vegetation and a large number of laser points that belong to artificial ground are misclassified into natural ground. For misclassified categories (vegetation and natural ground), reclassification will be carried out.

The superpixel segmentation is used for reclassification. In this paper, the PBA image is segmented into 2025 superpixel blocks. For each superpixel block, if the pixel proportion of vegetation is less than 1/8, the laser points corresponding to vegetation will be reclassified into the category with the highest pixel proportion in the block. If the superpixel block contains both natural ground and artificial ground, the laser points belonging to the category with a small proportion will be reclassified into that with a larger proportion. The images in the middle of Figure 15 and Figure 16 are the reclassification results and the images on the right of Figure 15 and Figure 16 show the ground truth. The recall rate and precision rate are given in Table 4 and Table 5.

After reclassification, the recall rates of vegetation and natural ground have been improved. However, for Testing Set A, the recall rate of low vegetation is still not high. A large number of laser points belonging to motorcycles are classified into low vegetation. Since motorcycles are not considered as a category, the lower recall rate is acceptable for low vegetation.

In addition, for Testing Set B, the precision rate of natural ground classification declined dramatically due to the disparity in the area between artificial ground and natural ground. After reclassification, some laser points belonging to natural ground are classified into artificial ground. Although this strategy sacrifices the precision rate of natural ground classification, it improves the precision rate of artificial ground classification greatly and the classification effect of the whole scene is better.

### 4.2. Classification Results of 3D Point Clouds Obtained in On-the-Fy Scanning Mode

In this subsection, a 3D laser point cloud dataset published by MINES ParisTech is selected to verify the algorithm. Since the data are obtained by on-the-fly scanning, pre-processing is performed to filter out some laser points with large errors. Simple cropping and downsampling are also performed to remove the laser points scanned into the interior of the building. A typical scene is selected for testing and the category distribution is shown in Table 6.

**Figure 17:**
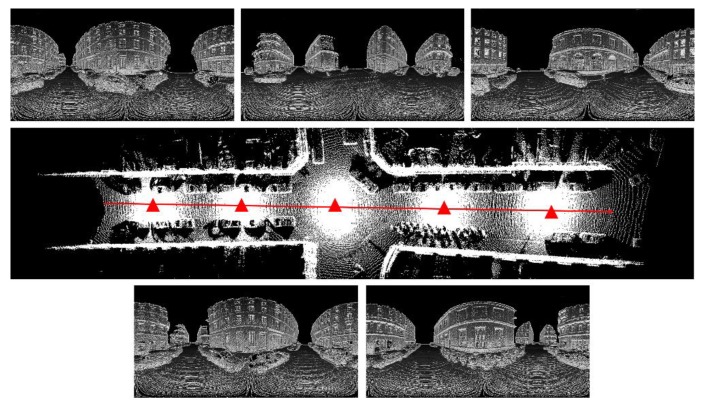
PBA images obtained from different viewpoints (**top** and **bottom**); 3D laser point cloud obtained by inverse mapping of PBA map (**middle**).

In on-the-fly scanning mode, multiple PBA images are needed to fully represent the 3D scene. As shown in Figure 17, the red ray approximates the trajectory of the data acquisition vehicle, and the length is about 80 m. The five red triangles are viewpoints selected on the acquisition trajectory. The images on the top and bottom of Figure 17 are the PBA images obtained from the five viewpoints.

**Figure 18:**
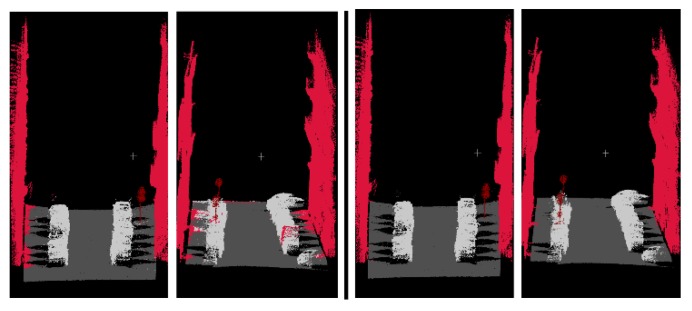
Point cloud classification results for MINES ParisTech Dataset (two images on the **left**) and the ground truth (two images on the **right**).

Due to the low density of data acquired by on-the-fly scanning, the resolution of the image at the bottom of the image pyramid is selected as 720 × 360 and FPFH features are not extracted. Since the scene contains only four categories, reclassification is not carried out. The classification results are shown in Figure 18.

We also compare the classification results with Weinmann’s work [14]. Weinmann selected a fixed neighborhood scale for point clouds and 21-dimensional features were extracted for each laser point. The comparison of classification results is shown in Table 7. It can be seen that the method proposed in this paper has obvious advantages for the classification of small objects such as railings and cars.

## 5. Conclusions

This paper presents an approach of 3D laser point cloud classification to accomplish outdoor scene understanding in urban environments. To improve the performance of point cloud classification, a new transformation model is proposed to transform point clouds to PBA images. Due to the correspondence between the original point cloud and the PBA image, multiple-scale features are extracted from both point clouds and PBA images, and then the Random Forest classifier is adopted to get initial classification results. To correct the misclassification points, reclassification is performed by remapping the classification results into the PBA images and using superpixel segmentation. Finally, we have conducted a series of experiments on two public datasets named ETH Zurich and MINES ParisTech, and testing results demonstrate the validity and the robustness of the proposed method.

## Figures and Tables

**Table 1:** The category distribution of the two testing sets.

	Artificial Ground	Natural Ground	High Vegetation	Low Vegetation	Building	Railing	Car
Testing Set A	3932832	0	30402	7685	4129066	492534	89467
Testing Set B	13663073	32956	276317	1326052	8755941	3499737	257352

**Table 2:** The classification evaluation metrics of Testing Set A.

	Artificial Ground	Natural Ground	High Vegetation	Low Vegetation	Building	Railing	Car
Precision	97.87%	-	79.08%	94.16%	94.41%	51.51%	89.07%
Recall	99.44%	-	20.72%	2.75%	98.86%	73.43%	91.90%

**Table 3:** The classification evaluation metrics of Testing Set B.

	Artificial Ground	Natural Ground	High Vegetation	Low Vegetation	Building	Railing	Car
Precision	81.28%	53.83%	76.33%	86.02%	84.49%	61.41%	71.66%
Recall	99.71%	0.7%	23.61%	57.61%	99.58%	87.12	12.57%

**Table 4:** The reclassification evaluation metrics of Testing Set A.

	Artificial Ground	Natural Ground	High Vegetation	Low Vegetation	Building	Railing	Car
Precision	97.95%	-	62.69%	96.27%	95.99%	71.59%	98.58%
Recall	99.48%	-	45.48%	5.27%	98.21%	77.58%	90.25%

**Table 5:** The reclassification evaluation metrics of Testing Set B.

	Artificial Ground	Natural Ground	High Vegetation	Low Vegetation	Building	Railing	Car
Precision	99.53%	2.02%	69.79%	84.56%	85.82%	61.41%	75.02%
Recall	99.49%	4.29%	30.75%	66.15%	99.46%	86.58	11.19%

**Table 6:** The category distribution of the testing set.

Artificial Ground	Building	Railing	Car
886,463	2,136,908	3912	232,714

**Table 7:** The comparison of classification results.

		Artificial Ground	Building	Railing	Car
Our results	Precision	93.80%	99.85%	76.76%	98.9%
	Recall	99.45%	98.32%	76.35%	91.45%
Weinmann’s results	Precision	96.4%	96.2%	5.5%	75.5%
	Recall	90.2%	95.7%	97.4%	60.6%
